# Antagonistic interaction networks are structured independently of latitude and host guild

**DOI:** 10.1111/ele.12235

**Published:** 2013-12-19

**Authors:** Rebecca J Morris, Sofia Gripenberg, Owen T Lewis, Tomas Roslin

**Affiliations:** 1 Department of Zoology, University of OxfordSouth Parks Road, Oxford, OX1 3PS, UK; 2 Section of Biodiversity and Environmental Research, Department of Biology, University of TurkuTurku, FI-20014, Finland; 3 Spatial Foodweb Ecology Group, Department of Agricultural Sciences, University of HelsinkiPO Box 27 (Latokartanonkaari 5), Helsinki, FI-00014, Finland

**Keywords:** Antagonistic network, guild, host-parasitoid, latitude, matrix size, network metrics, network structure, quantitative food web, specialisation, taxonomic diversity

## Abstract

An increase in species richness with decreasing latitude is a prominent pattern in nature. However, it remains unclear whether there are corresponding latitudinal gradients in the properties of ecological interaction networks. We investigated the structure of 216 quantitative antagonistic networks comprising insect hosts and their parasitoids, drawn from 28 studies from the High Arctic to the tropics. Key metrics of network structure were strongly affected by the size of the interaction matrix (i.e. the total number of interactions documented between individuals) and by the taxonomic diversity of the host taxa involved. After controlling for these sampling effects, quantitative networks showed no consistent structural patterns across latitude and host guilds, suggesting that there may be basic rules for how sets of antagonists interact with resource species. Furthermore, the strong association between network size and structure implies that many apparent spatial and temporal variations in network structure may prove to be artefacts.

## INTRODUCTION

For a wide range of taxa, species richness increases from the poles towards the equator (Rosenzweig 1995; Hillebrand 2004). Indeed, an increase in species richness with decreasing latitude is so ubiquitous that is has been described as one of the few fundamental “laws” in ecology (Lawton 1999). As a possible mechanism for the latitudinal gradient in species richness, ecologists have invoked a role for interspecific interactions such as competition, predation and parasitism. Such interactions are proposed to be more frequent, more intense and have stronger dynamic consequences in the tropics (Dobzhansky 1950; Janzen 1970; Schemske 2009; Schemske *et al.* 2009). Another factor suggested to contribute to high diversity in the tropics (and a potential consequence of high levels of interspecific interactions) is an increase in the specialisation of these interspecific interactions (Rohde 1992; Willig *et al.* 2003): where many species co-occur in the same community, niche partitioning – and thus specialisation – might be more pronounced.

Despite widespread assumptions about the presence and importance of latitudinal gradients in interspecific interactions, there have been relatively few attempts to assess them systematically (for a review, see Lewinsohn & Roslin 2008). In particular, evidence for latitudinal gradients in specialisation remains largely anecdotal. Two studies of interactions between herbivorous insects and their host plants reached contrasting conclusions about latitudinal trends in specialisation. While Novotny *et al*. (2006) found no evidence for differences in specialisation in tropical and temperate systems, Dyer *et al*. (2007) found a tendency for specialisation to increase towards the equator. Clearly, more such studies are needed; particularly ones that use a standardised framework to allow the integration and comparison of data sets collected using varying methods in widely spaced geographical locations.

One important approach to describing the structure and understanding the dynamics of interspecific interactions within ecological communities is the study of interaction networks (Ings *et al.* 2009). The structure of these networks may be critically linked to community stability and dynamics (Tylianakis *et al.* 2010). In addition, the network approach provides a standardised framework for quantifying levels of ecological specialisation (Blüthgen *et al.* 2006). Empirical studies increasingly generate information on not just the incidence, but the frequency of interspecific interactions within ecological networks (Memmott 2009), providing a richer and more nuanced description of their structure. To capture the key features of these quantitative interaction networks, ‘weighted’ network metrics have been developed (Bersier *et al.* 2002). As an advantage, such metrics are also less sensitive to sampling biases than their earlier qualitative counterparts (Bersier *et al.* 2002; Banasek-Richter *et al.* 2009). This facilitates standardised comparisons of networks from different environments, regions and latitudes.

Two recent papers have used quantitative interaction network metrics to compare mutualistic interaction networks across a latitudinal gradient. Dalsgaard *et al*. (2011) studied hummingbird-plant interactions across the Americas, finding greater specialisation at lower latitudes. In contrast, Schleuning *et al*. (2012), using a large global data set on flower visitation and seed dispersal interactions, found that specialisation was significantly lower at tropical than at temperate latitudes. Few studies have compared the structure of antagonistic networks over any large spatial scales – though variation in qualitative metrics characterising the structure of aquatic communities inhabiting pitcher plants has been documented across North America (Baiser *et al.* 2012). However, to our knowledge, no systematic assessment of changes in quantitative antagonistic networks with latitude has yet been attempted. As a sufficient number of quantitative antagonistic networks have now been compiled, the timing seems appropriate for a global analysis to be undertaken.

In this article, we examine latitudinal patterns in network structure for antagonistic bipartite networks, drawing on studies ranging from the High Arctic to the Tropics. We focus on networks involving insects and their parasitoids (i.e. parasitic insects that are free-living as adults, but which develop in or on the body of a single host individual). Using recent advances in quantitative network analysis (Bersier *et al.* 2002; Banasek-Richter *et al.* 2009; Dormann *et al.* 2009), we focus our assessment on network metrics reflecting both specialisation and emergent network structure. Crucially, we first explore potential biases introduced by the heterogeneity of the component data sets, arising from variations in the size of the networks (i.e. in the total number of interactions between individuals documented in the respective studies) and the taxonomic diversity of hosts involved. Having controlled for these sampling effects, we test the following hypotheses:

1. *Network specialisation increases with decreasing latitude*: Species-rich tropical communities have been proposed to comprise species that are more specialised than their temperate counterparts (Dyer *et al.* 2007; but see Novotny *et al.* 2002, 2006; Lewinsohn & Roslin 2008). If latitudinal patterns exist in specialisation at the level of individual species, such patterns should also be reflected in the specialisation of entire networks. Here, we test the hypothesis that specialisation of host-parasitoid networks is highest in the tropics.
2. *Network specialisation varies with the ecological guild of the component species*: For host-parasitoid networks, the biology of the host taxa involved is likely to influence the specificity of their interactions with other species through biological, ecological or behavioural host traits. For example, phytophagous insect guilds differ in terms of their specialisation (Novotny *et al.* 2010) because their feeding mode, developmental stage and feeding location affect which host species they can exploit. As hosts from different guilds are differentially detected and attacked by parasitoids, these differences are likely to translate into differences in parasitism rate, parasitoid species richness and parasitoid specificity (Hawkins 1994) – which in turn is expected to affect network-level metrics of specialisation and emergent network structure.

## MATERIALS AND METHODS

### Data set

Host-parasitoid network data sets were identified from the literature and from correspondence with researchers active within the subject field. We restricted our data set to 28 network studies that met a set of criteria (see Appendix S1) in terms of their taxonomic, spatial and temporal resolution. Studies were located in 19 countries across five continents spanning the latitudes 34.6° S to 74.5° N (decimal degrees) (Figure S1.1 in Appendix S1). The studies represented five insect host guilds: aphids, gallers, leaf chewers, leaf miners and trap nesters. Details of individual data sets are given in Appendix S1.

### Descriptors of network structure

To characterise network structure in a broad sense, we focused on standard metrics of quantitative bipartite network architecture as frequently used in cross-network comparisons (Tylianakis *et al.* 2007). More specifically, we examined metrics which either directly or indirectly reflect the degree of specialisation at the level of the network, and for which a specific prediction regarding changes with latitude could be made.

The metrics chosen were (1) weighted quantitative *linkage density,* (defined as the weighted diversity of interactions per species); (2) weighted quantitative *connectance* (the weighted realised proportion of possible links, calculated as quantitative linkage density divided by the number of species in the network); (3) weighted quantitative *generality* (the mean effective number of hosts per parasitoid weighted by their marginal totals); (4) weighted quantitative *vulnerability* (the mean effective number of parasitoids per host species, weighted by their marginal totals); and (5) quantitative weighted *modularity* (the degree to which a quantitative network can be divided into modules where within-module interactions are more prevalent than between-module interactions (Dormann & Strauss 2013; see Appendix S2 for equations for all metrics)). In addition, we used (6) the weighted quantitative *network specialisation index H2'*, which provides an alternative description of the degree of specialisation among hosts and parasitoids across an entire network (see Appendix S2 for equation; Blüthgen *et al.* 2006). As such, H2' quantifies the deviation between observed interaction frequencies and those expected if interaction frequencies were random; thus, it reflects the extent to which parasitoid species discriminate among the available host species.

Overall, lower values of weighted quantitative connectance, weighted quantitative linkage density, weighted quantitative generality and weighted quantitative vulnerability (hereafter connectance, linkage density, generality and vulnerability for brevity) indicate higher specialisation in the network (with generality and vulnerability focusing on specialisation at the level of parasitoids and hosts, respectively, and the other metrics at the level of the entire network). Conversely, high values of H2' and quantitative weighted modularity (hereafter modularity; both indexes ranging from 0 to 1) imply a high degree of specialisation at the network level.

Metrics were calculated in the *Bipartite* (version 2.01) package of R (Dormann *et al.* 2008, 2009), using the *empty.web=false* option to account for hosts present but not parasitised (see Appendix S2 for rationale). Since the specialisation index H2' is sensitive to the type of data (integer or non-integer), we used only the integer data (based on specific counts of interactions) for this particular network metric (the non-integer data set was too small for a meaningful analysis).

### Analyses

The relationships between each metric of specialisation and network size, taxonomic diversity (see below), latitude and host guild were examined through a set of generalised linear mixed models (outlined below). All statistical analyses were carried out using the *lme4* package (Bates *et al.* 2013) in the statistical computing environment *R* (R Core Team 2013). Where necessary, we log-transformed data to improve the normality of residuals. The *R* codes for the maximal linear mixed effects model for each analysis described below are specified in Appendix S2. The structure of the random effect component of our models was selected by comparing models with random intercept (with a different intercept for each Study) to those with a random intercept and slope (allowing, for each Study, for variation in the slope of the response depending on log-transformed web size), and selecting those with the lower Akaike Information Criteria (AIC) score (or AICc score as corrected for small sample size for Model Structures 3 and 5, Appendix S2; Burnham and Anderson 2002). Following the principle of parsimony, if there was minimal difference in AIC (< 1) between models, the simpler model was preferred. Following Crawley (2013), we simplified the maximal models by removing non-significant fixed effects until a minimum adequate model was obtained. P-values of fixed effects were obtained by likelihood-ratio tests of the full model with the explanatory variable included or excluded. We then refitted the minimum adequate model using Restricted Maximum Likelihood and visually inspected the residual plots to check for any obvious deviations from homoscedasticity or normality.

### Testing for the effects of matrix size on network structure

While quantitative network metrics are less prone to sampling effects than their qualitative counterparts, they may still be biased by sampling intensity (Blüthgen *et al.* 2008; Dormann *et al.* 2009). In our data set, the total number of interactions between individuals (i.e. the sum of interactions in a quantitative network matrix) varied by a factor of 1000 across the 28 studies (Table S1.1 in Appendix S1), reflecting variations in both sampling effort and in the abundance and detectability of interactions. For brevity, we refer to this value (the total number of interactions recorded between individuals) as the ‘matrix size’. As smaller sample sizes will likely overlook a larger proportion of interactions present, we predict that matrix size will be negatively correlated with metrics characterising the level of specialisation within networks (hereafter, metrics of network specialisation).

To control for the effects of matrix size we first analysed the relationship between matrix size and each of the six network metrics using linear mixed effects models (Model structure 1, Appendix S2). Each network metric (log transformed, apart from H2') was modelled as a function of the logarithm of matrix size. The structure of the random effect component of each model was chosen as described above. For connectance, we used a random intercept model to account for non-independence of networks from the same study. For the other five metrics the random intercept and slope models were used.

### Exploring reasons for relationship between matrix size and metrics

To explore the likely reasons for observed relationships between network metrics and matrix size (see Data set), we used techniques conceptually equivalent to rarefaction (Hurlbert 1971).

To examine whether the observed relationships between matrix size and network metrics were similar to those generated by sampling effects alone, we created smaller networks by randomly selecting a specified number of interactions (individual parasitism events) from each of the original networks. For each matrix size within a range from 2 to the full number of interactions within the network, we randomly picked a corresponding number of interactions. At each of these predefined matrix sizes, 100 replicate networks were created from each of the original networks through random subsampling. The mean value of each network metric for each matrix size (across 100 replicates, or 50 replicates for modularity) was then used as our response variable in further analyses. Since we explicitly wanted to examine the value of each metric vs. absolute sample size (rather than density), only networks based on integer values (176 networks from 17 studies; see Appendix S1) were included in the subsampling.

**Figure 1:**
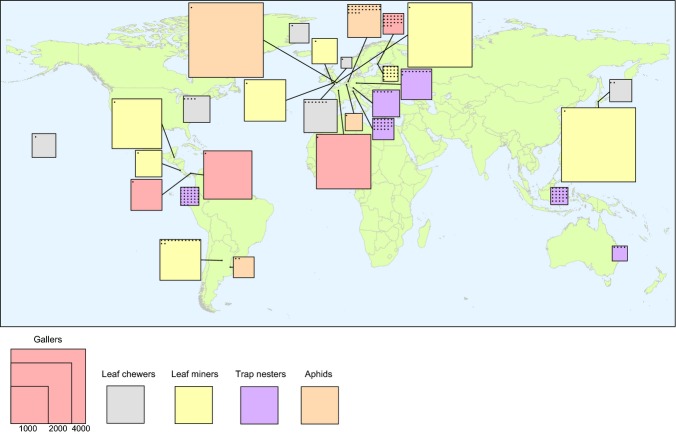
The location, matrix size and number of replicate host-parasitoid networks of each of 28 studies included in the analysis. Each study is depicted by a square, the size of which represents the logarithm of matrix size (i.e. the total sum of interactions in the respective quantitative network matrix). The number of dots within each square represents the number of replicate networks per study; for studies with more than one network, we show the mean size of individual networks. The colour of the squares represents the host guild targeted by each study as identified in the legend.

**Figure 2:**
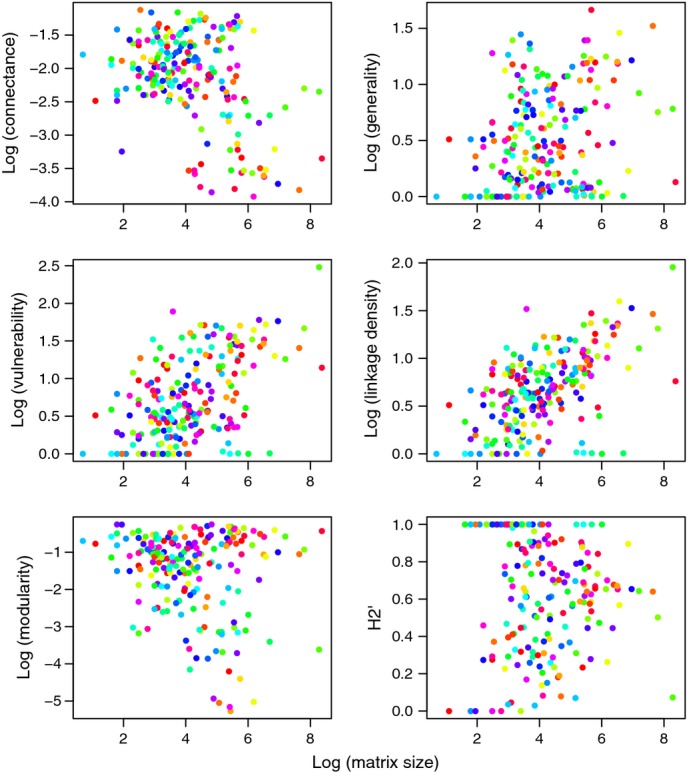
Logarithms of the six studied quantitative network metrics plotted against the logarithm of matrix size (i.e. the total sum of interactions in the respective quantitative network matrix). Networks from 28 individual host-parasitoid network studies are shown in different colours.

To test whether the relationships between matrix size and metrics that were evident in the original data (Fig.2) were also present when subsampling within individual networks, we then explored the relationships between network metric and matrix size by fitting linear regression models to the subsampled data. For each of our target metrics, we modelled the logarithm of the metric (mean value of the metric across 50 or 100 replicates; untransformed data were used for modularity and H2') as a function of the logarithm of the subsampled matrix size, in a random intercept model with the identity of the individual network nested within the study (Model structure 2, Appendix S2). For modularity, a random intercept and slope model was used (see Analyses above). If the original relationships between network metrics and matrix size at the level of full networks (Fig.1) were due to sampling effects alone, we predict that the relationships would also be repeated *within* individual networks following subsampling.

Finally, we tested whether the original relationships between matrix size and metrics were still evident *across* networks when subsampled to a standardised matrix size of 65 interactions (corresponding to the second tercile of empirical matrix size; see Appendix S3). To explore whether the relationships persisted when matrix size was standardised to 65 interactions, we regressed the logarithm of each metric (untransformed data for modularity and H2') from the standardised subsampled network (mean of 50 or 100 replicates) against the logarithm of the original matrix size, in a random intercept model (Model structure 3, Appendix S2). If the original patterns of relationships between network metrics and matrix size were due to sampling effects alone, we predict that such relationships would disappear following subsampling. In contrast, if the relationships are still present, processes other than pure sampling effects need to be invoked to explain the original pattern.

### Testing for the effects of host taxonomic diversity on network structure

A further potential source of bias in quantifying network structure arises from variations in the ‘completeness’ of different networks in terms of their taxonomic coverage. While some networks focus on a small range of closely related host species (e.g. Rott & Godfray 2000), others sample a much wider range of taxa (e.g. Lewis *et al.* 2002). The host preference of parasitoids is typically restricted to a rather limited set of phylogenetically related hosts (Desneux *et al.* 2012). Consequently, parasitoids in networks with high taxonomic diversity will tend to specialise on discrete sets of hosts, whereas parasitoids in networks with low taxonomic diversity, might be able to attack all or most of the host species present. Therefore, we predict that metrics of network specialisation will be higher in networks with greater taxonomic diversity.

To explore whether the observed relationships between matrix size and network metrics were due to larger networks encompassing taxonomically more diverse hosts with consequently fewer shared parasitoids, we explicitly assessed the taxonomic diversity of hosts included in each network, as described in Appendix S1. Using this information, we calculated the taxonomic diversity index, Δ, for the host species (Clarke & Warwick 1998). The calculations were implemented using the *taxondive* function in the R package vegan, using the option varstep = FALSE (Oksanen *et al.* 2013). We chose to focus on the taxonomy of the hosts (rather than the parasitoids) since sampling of host-parasitoid networks through rearing-based techniques is host-focused: researchers choose which hosts to sample, but cannot choose which parasitoids to include.

To investigate whether network metrics vary with host taxonomic diversity, we built linear mixed effects models of our focal network metrics (untransformed data for connectance and H2', log-transformed for the other metrics) as a function of Δ (Model structure 4, Appendix S2). A random intercept model was used for all metrics except generality, for which a random intercept and slope model was used (see Analyses above).

### Testing for effects of latitude and host guild on network structure

Given the observed relationships between matrix size and network metrics (see Data set), we adjusted for the large variation in matrix size by regressing the logarithm of each network metric (or untransformed data for H2') on the logarithm of matrix size, and then used the residuals from these regressions as our dependent variables in analyses testing for effects of host feeding guild and latitude. Using a linear mixed effects model approach, we modelled the residuals from each of these regressions as a function of host guild, latitude, Δ, and their two way interaction terms, simplifying the models as described above (Model structure 5, Appendix S2). In each case, we used models including a random intercept.

## RESULTS

### Effects of matrix size and host taxonomic diversity on network metrics

Consistent with our expectation that smaller matrix size results in higher network specialisation, generality, linkage density and vulnerability all increased significantly with matrix size (Table1; Fig.2; Table S3.1 in Appendix S3). Connectance significantly decreased with matrix size (Table1; Fig.2; Table S3.1 in Appendix S3), whereas H2' is designed to be scale independent (Blüthgen *et al.* 2006) and – consistent with expectation – did not change significantly with matrix size. Modularity, which is highly correlated with H2' (Dormann & Strauss 2013), was also not affected by matrix size.

**Table 1:** Likelihood ratio test results for regressions of quantitative network metrics on (a) matrix size for original networks; (b) matrix size for subsampled networks; and (c) taxonomic diversity Δ. The values highlighted in bold are statistically significant (*P* < 0.05).

	Matrix size (original networks)			Matrix size (subsampled networks)			Taxonomic diversity Δ		
	χ^2^	d.f.	*P*-value	χ^2^	d.f.	*P*-value	χ^2^	d.f.	*P*-value
Connectance	10.549	1	**0.001**	24903	1	**<0.001**	0	1	1
Generality	8.614	1	**0.003**	25367	1	**<0.001**	9.11	1	**0.003**
H2'	0.587	1	0.444	1.634	1	0.201	0.798	1	0.372
Linkage density	21.102	1	**<0.001**	29273	1	**<0.001**	14.42	1	**<0.001**
Modularity	1.729	1	0.189	24.04	1	**<0.001**	30.743	1	**<0.001**
Vulnerability	21.434	1	**<0.001**	24714	1	**<0.001**	17.86	1	**<0.001**

**Figure 3:**
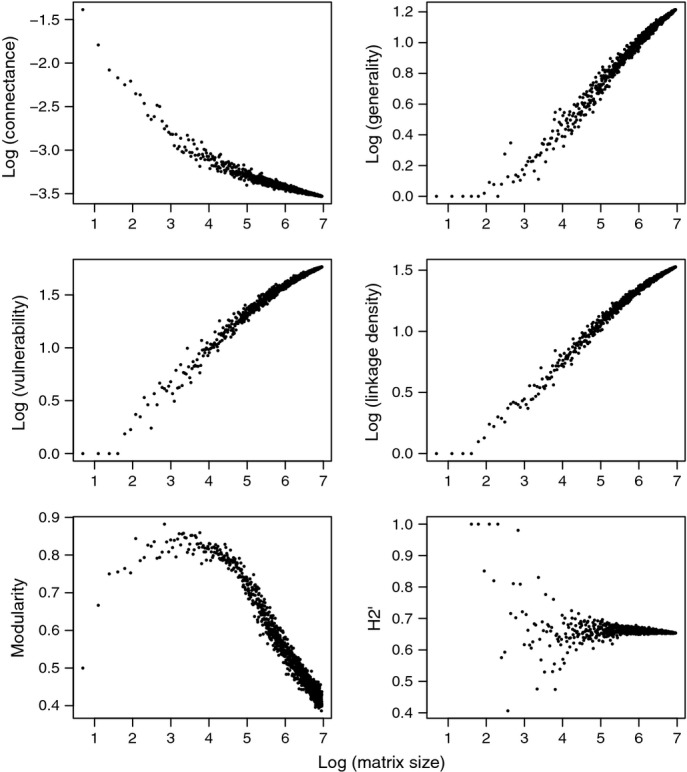
Quantitative metrics of network structure for subsampled interaction matrices plotted against the logarithm of matrix size. For clarity, we here show results for a single representative study (that of Lewis *et al.* 2002; for full results on all studies, see Figure S3.1, Appendix S3). For modularity and H2', we show untransformed data, whereas other metrics are shown on a log-scale. Each data point represents the mean of 50 or 100 replicate subsampled networks (see Materials and Methods for details).

For the subsampled data sets, regressions of network metrics (all log transformed apart from H2' and modularity) on the logarithm of matrix size showed patterns similar to those obtained in the analogous regressions on the original data (with modularity forming an exception; Table1; Fig.3; Table S3.2 and Fig. S3.1 in Appendix S3). This consistency suggests that the original relationships between network metrics and matrix size can be mostly attributed to sampling effects. As matrix size increased, connectance and modularity significantly decreased, while generality, vulnerability and linkage density significantly increased (Table1; Fig.3; Table S3.2 in Appendix S3). H2' did not vary detectably with matrix size.

Using the subsampled data sets with matrix size standardised to 65 interactions, regressions of the logarithm of the network metrics (all log transformed apart from H2' and modularity) on original matrix size were non-significant for all metrics – except for linkage density, which significantly increased with matrix size (χ^2^ = 5.92, d.f. = 1, *P *= 0.015). Thus, the original patterns between network metrics and matrix size appear to be primarily due to sampling effects, with little evidence of additional biological effects.

The regressions of network metrics against the taxonomic diversity index (Δ) revealed a significant effect of Δ on generality, vulnerability, linkage density and modularity (Table1). Networks with high taxonomic diversity showed higher generality and higher linkage density (reflecting low specialisation), higher modularity (reflecting high specialisation), and lower vulnerability (reflecting high specialisation), providing inconclusive evidence for the prediction that taxonomically more diverse networks are more specialised.

### Effects of latitude and host guild on network metrics

Residuals from regressions of metrics vs. matrix size and taxonomic relatedness were not detectably related to either latitude or guild (Table S3.3 in Appendix S3). Therefore, once we have accounted for the size and taxonomic diversity of networks, we can reject the hypotheses that specialisation increases with decreasing latitude, and that specialisation varies among guilds after controlling for latitude.

## DISCUSSION

Our study provides a first integrated analysis of structural patterns among networks of antagonistic interactions quantified in different parts of the world. Drawing on these diverse data, our explicit control of sampling effects allows us to critically evaluate hypotheses emerging from current theory on geographical patterns in species diversity and proposed trends in ecological specialisation. Overall, we found strong support for the prediction that network specialisation appears higher in studies focusing on smaller interaction matrices. There was less support for the prediction that taxonomically diverse networks will exhibit high levels of network specialisation: although significant changes in network structure were observed as taxonomic diversity of networks increased, the patterns detected were not consistently indicative of higher specialisation. Most surprisingly, we found no support for the hypotheses that network specialisation increases towards the tropics, or that – when controlling for latitude – specialisation within networks varies depending on the ecological guild of the component host species. In this regard, these antagonistic host-parasitoid networks seem to differ from networks of mutualistic interactions (Schleuning *et al.* 2012), suggesting that different processes may be moulding the architecture of antagonistic and mutualistic networks. Below, we discuss each of these findings in turn.

### Sampling issues in network studies

Both within and across the large number of antagonistic networks examined, we found a strong association between network size and measures of network structure. These patterns concerned network size in the sense of both the number of interactions examined and the taxonomic coverage of hosts. While previous network studies have addressed the importance of sampling intensity (*sensu* mean number of interactions per possible link in a network) and network dimensions (*sensu* number of species), they have not addressed the impact of the taxonomic diversity (Blüthgen *et al.* 2007, 2008; Dormann *et al.* 2009). Our approach of correcting for both matrix size and host taxonomic diversity allowed us to separate statistical patterns from underlying biology, revealing more evidence for the former.

Our findings come with important implications for network studies across habitats and regions. In recent years, there has been a marked increase in the number of studies involving the collection of replicated interaction networks between experimental treatments, or across ecological or land-use gradients (e.g. Schleuning *et al.* 2011; Devoto *et al.* 2012; and references in Supplementary Information). While the magnitude of variation in matrix size between replicates within individual studies is likely to be much smaller than among studies in our global data set, there is nevertheless a risk that methodological artefacts will affect the results. Some authors have controlled for differences in host abundance or host species richness among networks in their analyses (e.g. Tylianakis *et al.* 2007), but others have made uncontrolled comparisons. In future work, we propose that ecologists should control for potential effects of matrix size before conducting comparisons – just as ecologists comparing species richness between sites or treatments routinely standardise sampling effort using approaches such as rarefaction (Gotelli & Colwell 2001).

Standardising for effects of taxonomic diversity among networks may prove more problematic. For this reason, we suggest that any patterns emerging from studies integrating webs of highly differing taxonomic diversity should be supported by more in-depth comparisons between webs of similar taxonomic breadth. Alternatively, the effects of taxonomic breadth may be isolated by using descriptors such as Δ (the taxonomic diversity index; Clarke & Warwick 1998) as a covariate, or by simply subsampling taxa from larger networks before comparison to metrics from smaller networks. The latter approach will be similar to that proposed above for compensating for matrix size.

### Latitudinal patterns in network structure and specialisation

While communities vary tremendously in species richness and taxonomic complexity, our analysis of antagonistic interaction networks offers no evidence for consistent variation in network structure with latitude. This contrasts with the findings of Baiser *et al*. (2012) for aquatic networks inhabiting pitcher plants. Focusing on qualitative descriptors of network structure, these authors found increasing linkage density with increasing latitudes across North America – a pattern which they attributed to increasing species richness. However, for terrestrial insects, an often-assumed decrease in niche breadth (i.e. increasing diet specialisation) with decreasing latitude (Dyer *et al.* 2007) is expected to result in more specialised networks in the tropics. For this hypothesis, we found no support. Rather, the fact that network structure does not vary consistently with latitude suggests that there may be basic rules for how antagonists interact with each other across the globe, irrespective of the size and diversity of the networks that they comprise.

The lack of variation in the structure of the networks with latitude observed in our study across a global scale is consistent with the patterns observed by Kaartinen & Roslin (2011, 2012) at a landscape scale. These authors found pronounced spatiotemporal consistency in the structure of tens of host-parasitoid networks, despite major variation in both species richness and species identity in space and time. Thus, antagonistic networks may be similarly structured at both global and landscape scales.

Our findings from antagonistic networks contrast with reports of latitudinal patterns in the structure of mutualistic networks. Studying hummingbird-plant networks across the Americas, Dalsgaard *et al*. (2011) found greater biotic specialisation at lower latitudes. In contrast, Schleuning *et al*. (2012), using a global data set of quantitative mutualistic networks, found that biotic specialisation of mutualistic networks was significantly lower at tropical than at temperate latitudes. These authors attributed the pattern detected to a response of pollinators and seed dispersers at high latitudes to low plant diversity, since specialisation decreased with increased plant diversity. In our study, the same argument could be used to predict increasing specialisation of parasitoids towards the host-poor Arctic, but no such patterns were evident after correcting for matrix size and taxonomic diversity. We therefore suggest that host-parasitoid networks are structured by other forces – perhaps by basic constraints on the ability of parasitoids to locate diverse sets of hosts or to cope with their behavioural or immune responses.

Since Schleuning *et al*. (2012) used a metric designed to be insensitive to matrix size (Blüthgen *et al.* 2006), the discrepancy in latitudinal patterns among different types of interaction networks appears to be real rather than methodological. As such, it suggests that different forces structure networks of different interaction types. The specialisation of our host-parasitoid networks also appears much higher overall (mean H2' = 0.65, with a high s.d. of 0.31) than the equivalent values for mutualistic webs (Fig. 1c in Schleuning *et al.* 2012). Consequently, even if network specialisation does not change consistently with latitude for antagonistic networks, it may still be higher across antagonistic (specifically host-parasitoid) than mutualistic networks – regardless of latitude. A study by Poisot *et al*. (2011) found the opposite pattern, with mutualistic networks more specialised than antagonistic networks; although here, too, there was larger variation among the latter. However, the networks included in the study of Poisot *et al*. included antagonistic and mutualistic interaction types well beyond those included in the present study, and well beyond those studied by Schleuning *et al*. (2012). This lack of standardisation in interaction types may have contributed to the observed pattern.

Again, a general discrepancy between plant-pollinator or seed-disperser vs. host-parasitoid systems may be attributable to different constraints on resource selection. For parasitoids, host selection is likely to be affected by traits involved in host location and host defences, thus potentially restricting host use to fewer and more similar taxa than plants used by pollinators or seed dispersers.

At a general level, the idea that antagonistic and mutualistic networks may be structured differently is far from new. The stability of mutualistic networks is thought to be promoted by a highly connected and nested architecture, whereas stability in antagonistic networks is promoted by a compartmented and weakly connected structure (Thébault & Fontaine 2010; but see also James *et al.* 2012, 2013; Saavedra & Stouffer 2013). Highly connected networks will – by their nature – be characterised by lower specialisation than weakly connected antagonistic networks; as a consequence, networks of these two different types are likely to respond differently along a latitudinal gradient of species richness. If structure is related to stability, then the patterns unravelled here and by Schleuning *et al*. (2012) suggest that mutualistic networks may be more stable towards the tropics, whereas antagonistic networks (or at least those involving insect hosts and parasitoids) may be equally stable across latitudes.

### Network specialisation and insect host guilds

Across our data set, we found no evidence for network structure differing consistently among host guilds. This observation runs contrary to the expectation that the morphological and chemical defences of the host would affect specialisation at the level of parasitoids (Gauld *et al.* 1992; Hawkins 1994) and thus networks. Indeed, our analysis is among the first to address specialisation of parasitoids at the network level, but provides no evidence that specialisation in antagonistic networks is affected by the ecological guild of the host species, after accounting for variation in the size and diversity of the networks. In this context, we stress that the conclusions reached are conditional on the data examined: while our data set encompassed representatives of five major insect host guilds, analysing host-parasitoid networks across a wider range of host guilds might still reveal differences in network specialisation.

## CONCLUSIONS

In this article, we detected no relationship between the structure of host-parasitoid networks across different latitudes or as associated with different host taxa. Importantly, a lack of association between latitude and network structure does not imply a lack of variation, but that variation among latitudes is overridden by variation from other sources. The challenge now is to identify the dynamic processes generating this apparent uniformity in structure across a latitudinal gradient, amidst a haze of methodological complications. A significant concern to emerge from our analysis is that many spatial and temporal differences in network structure proposed to date may be largely indicative of methodological choices (i.e. of variation in the size and taxonomic diversity of the networks compared) rather than of biological processes. Methodological adjustments such as those used in this article allow these biases to be overcome – but relatively few data sets provide the quantitative data to allow this. While we wait for more studies of this kind, we urge ecologists to avoid uncontrolled comparisons across networks.
