# Supplementary material for: Antagonistic interaction networks are structured independently of latitude and host guild
Source: Ecol Lett. 2013 Dec 19;17(3):340–9. doi: 10.1111/ele.12235 (PMC4262010; doi:10.1111/ele.12235)
Supplement: Supplementary file 1 — Supplementary [file ele0017-0340-SD1.docx]

Supplementary information

**Appendix S1**

**Specifics of the data set**

Only data sets that fulfilled (or could be adjusted to fulfil) the following set of criteria were used. Specifically, networks had to a) contain the trophic levels of host and parasitoid (secondary parasitoids in aphid networks were combined with primary parasitoids and jointly treated as one trophic level); b) sample only one guild of hosts (sensu Novotny *et al.* 2010; for data sets with multiple host guilds we split the data into separate networks for each guild); c) record the numbers of hosts killed per parasitoid species (only parasitoids that attacked at least one host species were included); d) contain at least two host and two parasitoid species; and e) be collected during a time period of up to 12 months (if data were collected over a longer period, we randomly selected a 12-month period to be included in our study). Since we explicitly wanted to analyse networks in which all species could potentially interact in space, we analysed each replicate network within a study separately. (Aggregate networks spanning large areas would add artefacts in the form of species pairs which could not even hypothetically reach each other.) Our webs were well distributed across latitudes, matrix sizes, host guilds and taxonomic diversities (Δ; explained below and in the main paper; Figure S1.1). Host guilds were reasonably well dispersed across latitudes (Figure 1, main paper). In terms of latitude, we were interested in the effects of the distance from the equator (i.e. in potential differences between tropical and temperate sites), and did not include information on the hemisphere where study sites were located. Where a study included replicate networks, we used a study-specific latitude (typically median latitude) as an explanatory variable in our analyses, since for most studies the latitudes of the replicate networks were not available but likely close to each other.

For 17 of the studies, we had access to data matrices in the format of integer data (the number of hosts killed by each parasitoid species). For the remaining 11 studies interaction matrices showed the frequencies of interactions expressed in other (non-integer) units (e.g. the frequency of each interaction expressed per unit area). Matrix size in terms of the total number of interactions between individuals was obtained by summing all the interactions within the network matrix for integer data. For non-integer data, the number of interactions was either obtained from the original paper, or by multiplying the density of parasitoids (per m^2^) by the area sampled.

For calculating taxonomic diversity (Δ; Clarke & Warwick 1998), we used information in taxonomic databases (Beccaloni *et al.* 2003; Ratnasingham & Hebert 2007; Fauna Europaea 2012; Roskov *et al.* 2013) . Drawing on this information, we assigned each host species in the networks to the order, family, subfamily and genus level. The relatively few species with one or more unknown taxonomic levels were assigned taxonomic levels as far as possible (using e.g. Tortricinae_Gen, for a species of unknown genus in the Subfamily Tortricinae). This approach may have led to a slight underestimation of taxonomic diversity by lumping unknown taxonomic levels (in this case other Tortricinae_Gen) together, but such effects were only a potential problem for a few of the largest networks.

**Specifics of the data assembly for each study**

**Alhmedi et al. (2011)**

Host-parasitoid data for 2005 were obtained direct from the quantitative network figures in the publication, by measuring the width of the bars using Image J and adjusting these to densities using the scale shown in the original publication. Given that the network metrics used are based on proportions and as such are scale invariable, it is the relative densities that are important. Hence, any measurement error during the extraction procedure is unlikely to bias the resulting network metrics.

**Albrecht et al. (2007)**

The data sets were provided by the lead author. Interaction matrices were constructed for each habitat type (restored meadows or intensively managed grasslands) for each of 13 study sites. For each of the intensively managed grasslands, data from traps placed at different distances from the restored meadow were combined into one single matrix. For the intensively managed grasslands at sites 5 and 7, and for the restored meadow at site 10, the networks were too small for calculating network metrics of interest (<2 species of parasitoids). Hence, the total number of replicates was 23.

**Barbosa et al. (2007)**

Data were provided by Astrid Caldas. In the publication, data collected at different sites and throughout a period of several years were merged into single networks. To make the study comparable to other data points in our analysis, we focused on data collected in one randomly chosen year (1996). These data were collected at four sites (referred to as PAT, COL, CR, and FM in the data sets). Within these sites, we pooled data from different host plant species (box elder and black willow).

**Bukovinsky et al. (2008)**

Data used in the publication were provided by the lead author and by Frank van Veen. In addition they provided unpublished data on exact host aphid abundances. We recreated the 24 networks from the raw data using densities (counts per interaction per plant), as different numbers of plants were sampled on each of four sampling occasions. Primary and secondary parasitoids were pooled into one parasitoid trophic level.

**Cagnolo et al. (2009)**

The data used in Cagnolo et al. (2009) were obtained from the lead author. Data were collected over a period of two years. We constructed interaction matrices for each of the 14 study sites for the second year of the study (December 2002-March 2003). The data sets provided showed numbers of parasitoid individuals emerging from each of the host species. For most of the species, there is one individual emerging per host (L. Cagnolo, pers. comm.). To correct for multiple individuals emerging from each host individual in the parasitoid *Copidosoma* sp., we divided the number of parasitoid individuals by 8.5 (the average number of parasitoids emerging from each leaf miner host) for this species (L. Cagnolo, pers. comm.). In this way, we were able to obtain interaction matrices showing the approximate number of hosts killed by each parasitoid species. This number was rounded to the nearest integer (or, in cases where >0 but <1, rounded to 1).

**Carvalheiro et al. (2010)**

Data were extracted directly from the quantitative networks in the supplementary information of the paper by measuring bar lengths using Image J and adjusting to numbers of individuals using the scale bars provided. As explained above (under Alhmedi 2011), this method of obtaining the data is unlikely to bias the resulting network metrics. Only external feeders, not web spinners (hosts 4, 5 and 27) were included, and metrics were calculated for 8 external feeding networks. One hyperparasitoid (*Mesochorus* sp.) species was found, but this was excluded from the networks by the original authors.

**Clarke (2000)**

Data for one ancient woodland network for 1 field season (< 1 year) were provided by Jane Memmott. All hosts were leaf miner species. The site for this network was between Dursley in Gloucestershire and Porlock in Somerset. Since the exact location could not be reconstructed, we used the latitude corresponding to the median latitude between these two sites in our analyses.

**Gathmann et al. (1994) and Gathmann data *in* Tscharntke et al. (1998)**

Permission to use these data was given by Achim Gathmann. The data were collected in two areas (Karlsruhe and Göttingen), and hence provided two data points for our analyses.

***Karlsruhe data set:*** These data were collected in 1990 and are described in Gathmann et al. (1994) and Tscharntke et al. (1998). In the Tscharntke et al. paper, the data are referred to as ‘study 1’. While the original publication included many habitat types, the data given to us represented five habitat types: pea fields (PE), cereal fields (CE), Phacelia set aside fields (PH), set aside fields (SA) and orchard meadows (OM). (The original publication divided the SA into multiple types. In the data set provided to us, these had been pooled.) We were able to analyse interaction matrices for two of the habitat types: SA and OM. (The other habitat types either only had data on hosts – i.e. no parasitoids – or networks too small to allow calculation of the network metrics.) There were three replicates for each of these habitat types.

***Göttingen data set:*** The ‘Göttingen data’ are presented as ‘study 3’ in Tscharntke et al. (1998). We were given a subset of the data (material collected in 1996). These data represented five habitat types: chalk grasslands (CG), field margin strips (FM), extensively managed grasslands (GR), set aside fields (SA) and orchard meadows (OM). For four of these (CG, FM, GR and SA) the data sets were sufficiently large for us to calculate network statistics. For each of CG, FM and GR we were able to calculate network metrics for three replicate networks. For SA, only one replicate was sufficiently large for us to calculate network metrics.

**Hennemann and Memmott (2001)**

Data for 1 replicate site for 1 year (2000) were provided by Jane Memmott. Two carnivorous caterpillar species (*Eupithecia* sp.) were removed from the data set.

**Hirao & Murakami (2008)**

Data for the network presented in the article (Hirao & Murakami 2008) were provided by Masashi Murakami. We used the summary network (data pooled across the whole study period; Fig. 1 in the primary publication) as a data point in our study. Since the authors extrapolated from their sample of leaf miners per tree to the total number of leaf miners per tree, their network was many orders of magnitude larger than all our other networks. Therefore we chose to use the data from the original sample size before extrapolation. Since the network in the publication shows the number of parasitoid individuals that emerged from each host (rather than the number of hosts killed by respective parasitoid species), we corrected for this using information on multiple parasitism provided in the publication (p. 162). More specifically, we divided the number of *Achrysocharoides* sp. by three and the number of *Holcothorax* sp. by six. The data set provided by the author did not include information on host abundances. To be able to calculate the percentage of parasitism, approximate host densities were obtained by measuring the length of the bars in an enlarged version of Fig. 1 in the publication (using the software ImageJ). Since the length of the bars depicting the parasitoids could be calibrated using values in the interaction matrix (provided by the author), we were thus able to infer host abundances taking the conversion factor on Fig. 1 into account (parasitoid abundances are expressed at a scale 2.3× that of their hosts).

**Kaartinen and Roslin (2011)**

Data were provided by the lead author, who prepared the data for one year (2007) by dividing up her data into networks for leaf miners and for gallers, resulting in 22 replicate networks for each guild. Network metrics were calculated for 21 out of 22 of these networks for each guild (one network for each guild was too small to allow calculation of the metrics).

**Klein et al. (2006)**

Permission to use the data was given by the lead author. Data were already summarised for a 15 month period, and given that it would have been very time consuming for the lead author to summarise data for a 12 month period, we decided to use 15 months for this data set. Network metrics were calculated for all 24 replicate agroforest sites (differing in distance to nearest natural forest). Kleptoparasites were included as parasitoids.

**Lewis et al. (2002)**

Data for a one year summary network, as published, were provided by the lead author.

**MacFadyen et al. (2009)**

Data were provided by Jane Memmott, who provided a one year summary network for one of the 20 farms in the study. This single network was divided up into networks for leaf miners and external leaf chewers (we did not include semi-concealed feeders), with the assistance of Sarina MacFadyen, who provided details of the guilds of the unidentified/unreared species.

**Memmott et al. (1994)**

Permission to use the data was obtained from Jane Memmott and the data were provided by Charles Godfray. Only data from the quantitative sampling were used (one year), resulting in 62 hosts and 39 parasitoid species. This is fewer parasitoid species than shown in the quantitative network in the paper, however, we believe that the extra parasitoid species shown in the paper were obtained from additional sampling.

**Morris (unpublished data)**

Data were collected on cavity nesting hymenopterans and their parasitoids using trap nests by Rebecca Morris and Frazer Sinclair in Lamington National Park, Queensland, Australia between December 2006 and April 2007. Host-parasitoid interactions were documented by rearing. Data used were for 4 replicate quantitative networks collected at an elevation of approximately 300 m over 5 months. Unidentified species (*Fabriogenia* sp. and *Pison* sp.; 3% of host individuals) were excluded.

**Muller et al. (1999)**

Permission to use the data was obtained by Charles Godfray and the data provided by Frank van Veen. The data set may be slightly different to the original Muller et al. 1999 paper due to subsequent improvements in parasitoid identification. The data set for year 1995 (selected randomly) was used for the analyses. Both primary parasitoids and secondary parasitoids were included as “parasitoids”, and were linked directly to aphid species.

**Murakami et al. (2008)**

Data used in the original publication were provided by the lead author. Networks were constructed separately for each of two study sites (refered to as the ‘high’ and ‘low’ density sites in the publication). To create networks with only free feeding hosts, the three leafminer hosts (*Stigmella* sp., *Phyllonorycter* sp. and *Rhynchaenus japonicus*) and their associated parasitoid species were removed from the data set. To avoid redundant data points, we did not construct and analyse leaf miner networks. (The study by Hirao and Murakami – also included in our meta-analysis – was conducted at the same site and concerns leaf miners.) To be able to calculate parasitism rates, total host densities for each study site were obtained from Table 2 in the original publication. Comparing information in this table with information on host abundances in Table 6 suggests that the values for the two sites have by mistake been swapped. Hence, we assumed that the total host density was 521 at the high density site and 263 at the low density site.

**Omacini et al. (2001)**

Data were obtained from Enrique Chaneton for summary networks for the 20 replicates in each treatment (endophyte+ and endophyte -) as shown in the paper.

**Paniagua et al. (2009)**

We used the same data sets as in the original publication (Paniagua et al. 2009), co-authored by one of us. Permission to use the data in this new context was obtained from the lead author. For one of the study sites (Parque Natural Metropolitano), we rearranged the data by combining material collected at different strata (canopy and understory) into one interaction matrix. Since the two sites in the original publication are separated by 80 km and differ markedly in their rainfall regime and plant species composition, they were considered to be two separate studies in our analysis.

**Roslin & Várkonyi (unpublished data)**

The data were collected by Tomas Roslin and Gergely Várkonyi at the Zackenberg research station, Northeast Greenland (74°30'N 21°00'W) between 5 June and 6 July, 2010. Sampling was specifically focused on larvae of Lepidoptera and their associated parasitoids. To obtain quantitative information on host abundances, hosts were obtained by sweep-netting, visual search and live-trapping pitfalls within an area of ca 10 km^2^ (for details see Várkonyi & Roslin 2013). Host-parasitoid interactions were documented by rearing. The data set includes information on 428 host individuals of 11 species and 46 parasitoid individuals of 8 species. Despite the data being collected on a slightly larger spatial scale than in other studies, this data set was treated as one network, given the open nature of the tundra landscape and the dispersal abilities of the large lepidopterans and parasitoids involved.

**Rott & Godfray (2000)**

The published data were provided by Charles Godfray. One year (1993) was selected at random and the two generations of data from that year were added together to create a one year summary.

**Sinclair (2012)**

The data provided by Frazer Sinclair show rearings of Cynipid galls collected in 2009 during quantitative surveys of an experimental plantation of *Quercus petraea* in the forest of Petite Charnie, Sarthe, Northwest France. Sexual and asexual generations of individual gall-inducer species were considered to be distinct types. Sexual generations were surveyed and collected during May-June, asexual generations during August-October. Emerging inquilines (Hymenoptera: Cynipidae: Synergini) were excluded. The interaction matrix given to us showed the total number of parasitoid individuals emerged for each host×parasitoid species combination. To make the data set comparable to other data sets in our analysis, we modified the matrix to show the total number of host individuals killed for each host×parasitoid species combination. Using a separate data set on galls that had been reared individually (provided by Frazer Sinclair), we assessed the mean number of parasitoids emerging from individual hosts for each host×parasitoid combination. We then divided the entries in the original matrix by these numbers, rounded the obtained values to the nearest integer and used this corrected matrix when calculating network metrics. (In the majority of cases, the ratio of parasitoids to hosts was 1 or very close to 1, making the modified matrix very similar to the original matrix.)

**Tylianakis et al. (2007)**

Data for a 17 month period were provided by Jason Tylianakis. We selected the first 12 month period (November 2003 – October 2004) for our analysis. Network metrics were calculated for each replicate within each of 5 habitat types (forest, abandoned coffee agroforest, coffee agroforest, pasture and rice). Forest and abandoned agroforest had 6 replicates each and the other 3 habitat types had 12 replicates. Of these a number of replicates showed networks too small to calculate metrics, so metrics were calculated for 39 replicates in total.

**Table S1.1.** Details of the network studies included in the analyses. For studies with more than one replicate network, values represent means for number of host species, parasitoid species, matrix size (i.e. the total sum of interactions in a quantitative network matrix) and Δ; and medians for latitude. Asterisked studies indicate integer data.

| **Study reference** | **Host guild** | **Country** | **Latitude**  **(D.Dº)** | **Number of**  **replicate networks** | **Temporal extent**  **of data used** | **Number of**  **host species** | **Number of**  **parasitoid species** | **Total number**  **of species** | **Matrix size**  **(total number of interactions among individuals)** | **Δ**  **(taxonomic diversity)** |
| --- | --- | --- | --- | --- | --- | --- | --- | --- | --- | --- |
| *Albrecht et al. 2007 | trap nesters | Switzerland | 47.517 | 23 | 12 months | mean 8.068 | mean 4.549 | mean 12.617 | mean 60.905 | 44.453 |
| Alhmedi et al. 2011 | aphids | Belgium | 50.563 | 1 | 12 months | 5 | 6 | 11 | 30.06 | 24.026 |
| *Barbosa et al. 2007 | leaf chewers | USA | 39.05 | 4 | 12 months | mean 52.75 | mean 27 | mean 79.75 | mean 123.5 | 66.484 |
| Bukovinszky et al. 2008 | aphids | Netherlands | 51.95 | 24 | 1 field season | mean 2 | mean 15 | mean 17 | mean 179.94 | 2.651 |
| *Cagnolo et al. 2009 | leaf miners | Argentina | -31.219 | 14 | 1 field season | mean 64.57 | mean 59.286 | mean 123.856 | mean 550.857 | 78.54 |
| Carvalheiro et al. 2010 | leaf chewers | UK | 50.817 | 8 | 1 field season | mean 9.875 | mean 5.5 | mean 15.375 | mean 13.57 | 40.293 |
| Clarke 2000 | leaf miners | England | 51.445 | 1 | 1 field season | 23 | 16 | 39 | 108.835 | 65.855 |
| *Gathmann  *in* Tscharntke et al. 1998 | trap nesters | Germany (Gottingen) | 51.534 | 10 | 1 field season | mean 13.083 | mean 6.583 | mean 21.416 | mean 173.5 | 44.743 |
| *Gathmann et al. 1994 | trap nesters | Germany (Kalrsruhe) | 49.017 | 6 | 1 field season | mean 8.333 | mean 6.667 | mean 15.5 | mean 133.83 | 48.185 |
| *Henneman & Memmott 2001 | leaf chewers | Hawaii | 22.14 | 1 | 12 months | 26 | 9 | 35 | 90 | 37.537 |
| Hirao & Murakami 2008 | leaf miners | Japan | 42.717 | 1 | 1 field season | 16 | 58 | 74 | 3944.01 | NA |
| *Kaartinen et al. 2011 | leaf miners | Finland | 60.183 | 21 | 12 months | 6.81 | mean 4.476 | mean 11.286 | mean 20.048 | 42.585 |
| *Kaartinen et al. 2011 | gallers | Finland | 60.183 | 21 | 12 months | 7.333 | mean 6.19 | mean 13.523 | mean 57 | 15.916 |
| *Klein et al. 2006 | trap nesters | Indonesia | -1.4188 | 24 | 15 months | mean 6.08 | mean 6.02 | mean 12.1 | mean 29.833 | 38.48 |
| *Lewis et al. 2002 | leaf miners | Belize | 16.733 | 1 | 12 months | 93 | 99 | 192 | 1053 | 77.297 |
| *Macfadyen et al. 2009 | leaf chewers | UK | 51.367 | 1 | 12 months | 33 | 30 | 63 | 573 | 66.327 |
| *Macfadyen et al. 2009 | leaf miners | UK | 51.367 | 1 | 12 months | 18 | 11 | 29 | 18 | 40.788 |
| Memmott et al. 1994 | leaf miners | Costa Rica | 10.883 | 1 | 12 months | 62 | 39 | 101 | 125 | NA |
| *Morris unpubl. | trap nesters | Australia | -28.217 | 4 | 1 field season | mean 10.5 | mean 5 | mean 15.5 | mean 18.75 | 47.244 |
| Müller et al. 1999 | aphids | UK | 51.4 | 1 | 12 months | 26 | 36 | 62 | 4312.37 | NA |
| *Murakami et al. 2008 | leaf chewers | Japan | 42.716667 | 2 | 12 months | mean 51.5 | mean 11.5 | mean 63 | mean 74.5 | NA |
| Omacini et al. 2001 | aphids | Argentina | -34.597 | 2 | 1 month | mean 2 | mean 6.5 | mean 8.5 | mean 58.045 | 12.100 |
| Paniagua et al. 2009 | gallers | Panama (APSL) | 9.283 | 1 | 12 months | 11 | 22 | 33 | 216 | NA |
| Paniagua et al. 2009 | gallers | Panama (PNM) | 8.966667 | 1 | 12 months | 21 | 40 | 61 | 946 | NA |
| *Roslin & Várkonyi unpubl. | leaf chewers | Greenland | 74.5 | 1 | 12 months | 8 | 8 | 16 | 46 | 59.080 |
| Rott & Godfray 2000 | leaf miners | UK | 51.4 | 1 | 12 months | 12 | 25 | 37 | 2443.54 | 12.755 |
| *Sinclair 2012 | gallers | France | 48.09 | 1 | 1 field season | 17 | 23 | 40 | 1333 | 19.63 |
| *Tylianakis et al. 2007 | trap nesters | Ecuador | -1.55 | 39 | 12 months | mean 11.3 | mean 2.392 | mean 13.692 | mean 38.092 | 54.557 |

**Figure S1.1.** Frequency histograms of host-parasitoid networks in the data set as partitioned by a) latitude; b) log (matrix size) (i.e. the total sum of interactions in the respective quantitative network matrix); c) insect host guild and d) taxonomic diversity (Δ).

**Appendix S2**

**Quantitative network metrics**

Weighted quantitative versions of network metrics were calculated following Bersier et al. (2002), Blüthgen et al. (2006), Dormann et al. (2009), Dormann and Strauss (2013) and Tylianakis et al. (2007). None of the measures of specialisation account for phylogenetic relationships or ecological similarity among species; they assume that all species can adjust their interactions according to the availability of partners, irrespective of morphological, behavioural or spatio-temporal constraints (Blüthgen 2006). Metrics were calculated in the *Bipartite* (version 2.01) package of R (Dormann *et al.* 2008; Dormann *et al.* 2009), using the *empty.web=false* option to account for hosts present but not parasitised. Host-parasitoid networks are defined by the host guilds sampled, and since all host species of commonly studied host-parasitoid networks (e.g. aphids and leaf miners) can be parasitised by at least one parasitoid across space or time, such unparasitised species can be considered an integral part of each focal community. The apparent absence of parasitism for some hosts will typically reflect low abundances and sample sizes for individual species. Yet, with the exception of connectance, we found that excluding unparasitised hosts did not alter the weighted metrics in practice.

The equations of the individual metrics, which are given below, include the following terms:

*I*  number of species at the lower trophic level

*J* number of species at the higher trophic level

*m* total number of interactions for all species

a*_ij_* number of interactions between species *i* from the lower trophic level and species *j* from the higher trophic level

A*_i_* total number of interactions of species *i* from the lower trophic level

A*_j_* total number of interactions of species *j* from the higher trophic level

H*i* the Shannon diversity of interactions for lower trophic level species:

$$H_{i}= -\sum_{j=1}^{J} \left( \frac{a_{ji}}{A_{i}} .\ln\frac{a_{ji}}{A_{i}} \right)$$

H*j* the Shannon diversity of interactions for higher trophic level species:

$$H_{j}= -\sum_{i=1}^{I} \left( \frac{a_{ij}}{A_{j}} .\ln\frac{a_{ij}}{A_{j}} \right)$$

***Weighted quantitative generality (G_qw_) -*** reflects the mean effective number of hosts per parasitoid weighted by their marginal totals. Thus, it was calculated as:

$$G_{qw}=\sum_{j=1}^{J} \frac{A_{j}}{m}2^{H_{j}}$$

***Weighted quantitative vulnerability (V_qw_) -*** reflects the the mean effective number of parasitoids per host species, weighted by their marginal totals. It is analogous to generality but with *j* replaced by *i* and *J* by *I* in the equation above:

$$V_{qw}=\sum_{i=1}^{I} \frac{A_{i}}{m}2^{H_{i}}$$

***Weighted quantitative linkage density (LD_qw_)*** – reflects the weighted diversity of interactions per species, and is calculated as the mean of G_qw_ and V_qw_:

$${LD}_{qw}= \frac{1}{2}\left( \sum_{j=1}^{J} \frac{A_{j}}{m}2^{H_{j}}+ \sum_{i=1}^{I} \frac{A_{i}}{m}2^{H_{i}} \right)$$

***Weighted quantitative connectance* *(C_qw)_*** – reflects the weighted realised proportion of possible links, calculated as:

$$C_{qw}= \frac{{LD}_{qw}}{s}$$

where *LDq* is the weighted quantitative linkage density, and *s* is the number of species in the network (including un-parasitised host species) (Tylianakis *et al.* 2007).

***Weighted quantitative modularity (Q) –*** describes the degree to which a quantitative network can be divided into modules where within-module interactions are more prevalent than between-module interactions, with module boundaries defined using an algorithm based on hierarchical random graphs (Dormann & Strauss 2013). Calculated as:

$$Q= \frac{1}{2N} \sum_{ij} \left( A_{ij}- K_{ij} \right)\delta\left( m_{i}, m_{j} \right)$$

where *N* is the total number of observed interactions in the network and A*ij* is the normalised observed number of interactions between *i* and *j*. The expected value, based on an appropriate null model, is given in the matrix *K*. The module to which a species *i* or *j* is assigned is *m_i_*, *m_j_*. The indicator function *δ* (*m_i_*;*m_j_*) = 1 if *m_i_* = *m_j_* and 0 if *m_i_* ≠ *m_j_*. *Q* ranges from 0 (which means the community has no more links within modules than expected by chance) to a maximum value of 1. Occasionally, bipartite was unable to successfully compute a modularity value, particularly for small webs; in these cases (<0.3% of attempts) mean values for modularity were based on fewer than 50 replicates.

***H2’*** – provides a description of the degree of specialisation among hosts and parasitoids across an entire network. Also termed the weighted quantitative *network specialisation index*, *H2’* is calculated as:

$$H_{2}^{'}= \frac{H_{2 max}- H_{2}}{H_{2 max}- H_{2 min}}$$

Where *H*_2_ is:

$$H_{2}= - \sum_{i=1}^{r} \sum_{j=1}^{c} \left( p_{ij}.\ln p_{ij} \right)$$

and

$$p_{ij}=\frac{a_{ij}}{m}, \mathrm{where} \sum_{i=1}^{r} \sum_{j=1}^{c} p_{ij}=1$$

and *r* and *c* are the rows and columns of the interaction matrix, respectively (see Blüthgen 2006 for further details).

**Appendix S3**

**Added details on statistical models and results**

To allow the replication of analyses conducted in the paper, we here offer the R-syntax used to fit the maximal linear mixed effects models (i.e. the models with all terms included before model reduction; see main text). In most cases, our response variables were log-transformed prior to analysis, and the models shown will therefore relate to log-transformed metrics of network structure. Each model was fitted separately to each of six metrics. All analyses were implemented in R, using the lme4 package.

**Model structure 1: Regression of network metrics on matrix size for original networks:**

Random intercept model:
Model1 <- lmer (log (metric) ~ log (matrix_size) + (1 | Study))

Random intercept and slope model:
Model1 <- lmer (log (metric) ~ log (matrix_size) + (1 + log (matrix_size) | Study))

**Model structure 2: Regressions of network metrics on matrix size for subsampled data set:**

Random intercept model:
Model2 <- lmer (log (metric) ~ log (matrix_size) + (1 | Study / Replicate))

Random intercept and slope model:
Model2 <- lmer (log (metric) ~ log (matrix_size) + (1 + log (matrix_size) | Study / Replicate))

**Model structure 3: Regression of network metrics on original matrix size for standardised matrix size (65 interactions):**

Model3 <- lmer (log (metric) ~ log (original_matrix_size) + (1 | Study))

**Model structure 4: Regressions of network metrics on taxonomic diversity (Δ):**

Model4 <- lmer (log (metric) ~ Δ + (1 | Study))

**Model structure 5: Regressions of residuals from Model 1 on latitude and guild**

Model5 <- lmer (metric_residuals ~ guild + latitude + Δ + guild:latitude + latitude:Δ + guild:Δ + (1 | Study))

**Table S3.1.** Regression coefficients from models of the logarithm of quantitative network metrics (Model structure 1; for likelihood ratio test results see Table 1, main text). The values highlighted in bold are statistically significant (p < 0.05).

| **Metric** | **Coefficient** | **Estimate** | **s.e.** | **t** | **p** |
| --- | --- | --- | --- | --- | --- |
|  |  |  |  |  |  |
| Connectance | Intercept | -2.193 | 0.176 | -12.465 |  |
|  | Log (matrix size) | -0.074 | 0.023 | -3.272 | **0.001** |
| Generality | Intercept | 0.092 | 0.131 | 0.702 |  |
|  | Log (matrix size) | 0.096 | 0.028 | 3.426 | **0.003** |
| H2’ (integer data) | Intercept | 0.779 | 0.171 | 4.549 |  |
|  | Log (matrix size) | -0.027 | 0.034 | -0.792 | 0.444 |
| Linkage density | Intercept | 0.076 | 0.111 | 0.687 |  |
|  | Log (matrix size) | 0.141 | 0.025 | 6.007 | **<0.001** |
| Modularity | Intercept | -0.789 | 0.219 | -3.599 |  |
|  | Log(matrix size) | -0.086 | 0.058 | -1.473 | 0.189 |
| Vulnerability | Intercept | 0.082 | 0.107 | 0.747 |  |
|  | Log (matrix size) | 0.159 | 0.026 | 6.195 | **<0.001** |

**Table S3.2.** Regression coefficients from models of the logarithm of selected quantitative network metrics (Model structure 2; for likelihood ratio test results see Table 1, main text). The values highlighted in bold are statistically significant (p < 0.05).

| **Metric** | **Coefficient** | **Estimate** | **s.e.** | **t** | **p** |
| --- | --- | --- | --- | --- | --- |
|  |  |  |  |  |  |
| Connectance | Intercept | -1.300 | 0.110 | -11.78 |  |
|  | Log (matrix size) | -0.202 | 0.001 | -223.75 | **<0.001** |
| Generality | Intercept | -0.072 | 0.061 | -1.17 |  |
|  | Log (matrix size) | 0.171 | 0.001 | 27.53 | **<0.001** |
| H2’ (integer data) | Intercept | 0.681 | 0.083 | 8.188 |  |
|  | Log (matrix size) | 0.007 | 0.022 | 0.330 | 0.201 |
| Linkage density | Intercept | 0.115 | 0.053 | 2.161 |  |
|  | Log (matrix size) | 0.131 | 0.017 | 7.676 | **<0.001** |
| Modularity | Intercept | 0.49 | 0.019 | 26.0 |  |
|  | Log (matrix size) | -0.043 | 0.007 | -5.98 | **<0.001** |
| Vulnerability | Intercept | -0.031 | 0.069 | -0.44 |  |
|  | Log (matrix size) | 0.178 | 0.001 | 222.29 | **<0.001** |

**Table S3.3.** Likelihood ratio test results for linear models of network metrics (model structure 5). Here, we used residuals from the regressions of logarithms of selected quantitative network metrics on logarithms of matrix size as our response variable, and the taxonomic diversity index, Δ, as a covariate. No significant effects of latitude or guild were found. The values highlighted in bold are statistically significant (p < 0.05).

| **Response** | **Explanatory variable** | **χ ^2^** | **df** | **p** |
| --- | --- | --- | --- | --- |
|  |  |  |  |  |
| Connectance | Guild : Latitude | 0.2434 | 4 | 0.993 |
|  | Guild : Δ | 3.608 | 4 | 0.462 |
|  | Latitude : Δ | 0.2364 | 1 | 0.627 |
|  | Δ | 0 | 1 | 1 |
|  | Guild | 0.733 | 4 | 0.947 |
|  | Latitude | 0.044 | 1 | 0.834 |
|  |  |  |  |  |
| Generality | Guild : Latitude | 1.031 | 4 | 0.905 |
|  | Guild : Δ | 6.297 | 4 | 0.178 |
|  | Latitude : Δ | 0.013 | 1 | 0.909 |
|  | Δ | 3.876 | 1 | 0.049 |
|  | Guild | 4.615 | 4 | 0.329 |
|  | Latitude | 0.219 | 1 | 0.64 |
|  |  |  |  |  |
| H2’ | Guild : Latitude | 0.158 | 3 | 0.984 |
|  | Guild : Δ | 1.454 | 3 | 0.693 |
|  | Latitude : Δ | 0.049 | 1 | 0.824 |
|  | Δ | 0.166 | 1 | 0.684 |
|  | Guild | 0.370 | 3 | 0.946 |
|  | Latitude | 1.446 | 1 | 0.229 |
| Linkage Density | Guild : Latitude | 1.161 | 4 | 0.885 |
|  | Guild : Δ | 8.045 | 4 | 0.090 |
|  | Latitude : Δ | 1.383 | 1 | 0.124 |
|  | Δ | 0 | 1 | 1 |
|  | Guild | 2.127 | 4 | 0.712 |
|  | Latitude | 0.176 | 1 | 0.675 |
|  |  |  |  |  |
| Modularity | Guild : Latitude | 2.734 | 4 | 0.603 |
|  | Guild : Δ | 6.150 | 4 | 0.188 |
|  | Latitude : Δ | 0.008 | 1 | 0.928 |
|  | Δ | 7.673 | 1 | **0.006** |
|  | Guild | 1.669 | 4 | 0.796 |
|  | Latitude | 0.551 | 1 | 0.458 |
| Vulnerability | Guild : Latitude | 1.693 | 4 | 0.792 |
|  | Guild : Δ | 9.537 | 4 | 0.049 |
|  | Latitude : Δ | 0.454 | 1 | 0.500 |
|  | Δ | 0 | 1 | 1 |
|  | Guild | 0.906 | 4 | 0.924 |
|  | Latitude | 0.643 | 1 | 0.423 |

**Figure S3.1.** Quantitative network metrics plotted against the logarithm of sub-sampled matrix size (the total sum of interactions in the respective quantitative network matrix) for 176 networks from 17 studies. For modularity and H2’, we show untransformed data, whereas other metrics are shown on a log-scale. Each data point represents the mean of 50 or 100 replicate sub-sampled networks. See main paper for details.

**References**

1.

Albrecht, M., Duelli, P., Schmid, B. & Muller, C.B. (2007). Interaction diversity within quantified insect food webs in restored and adjacent intensively managed meadows. *Journal of Animal Ecology*, 76, 1015-1025.

2.

Alhmedi, A., Haubruge, E., D'Hoedt, S. & Francis, F. (2011). Quantitative food webs of herbivore and related beneficial community in non-crop and crop habitats. *Biol. Control*, 58, 103-112.

3.

Barbosa, P., Caldas, A., Charles, H. & Godfray, J. (2007). Comparative food web structure of larval macrolepidoptera and their parasitoids on two riparian tree species. *Ecological Research*, 22, 756-766.

4.

Beccaloni, G., Scoble, M., Kitching, I., Simonsen, T., Robinson, G., Pitkin, B.*, et al.* (2003). The Global Lepidoptera Names Index (LepIndex)

Available at: <http://www.nhm.ac.uk/entomology/lepindex>, last accessed 09 Sept 2013

5.

Bersier, L.F., Banasek-Richter, C. & Cattin, M.F. (2002). Quantitative descriptors of food-web matrices. *Ecology*, 83, 2394-2407.

6.

Blüthgen, N., Menzel, F & Blüthgen, N (2006). Measuring specialization in species interaction networks. *BMC Ecology*, 6, 9.

7.

Bukovinszky, T., van Veen, F.J.F., Jongema, Y. & Dicke, M. (2008). Direct and indirect effects of resource quality on food web structure. *Science*, 319, 804-807.

8.

Cagnolo, L., Valladares, G., Salvo, A., Cabido, M. & Zak, M. (2009). Habitat fragmentation and species loss across three interacting trophic levels: effects of life-history and food-web traits. *Conserv. Biol.*, 23, 1167-1175.

9.

Carvalheiro, L.G., Buckley, Y.M. & Memmott, J. (2010). Diet breadth influences how the impact of invasive plants is propagated through food webs. *Ecology*, 91, 1063-1074.

10.

Clarke, M. (2000). The impact of habitat fragmentation on community processes. PhD thesis. University of Bristol, Bristol.

11.

Dormann, C.F., Fründ, J., Blüthgen, N. & Gruber, B. (2009). Indices, graphs and null models: analyzing bipartite ecological networks. *The Open Ecology Journal*, 2, 7-24.

12.

Dormann, C.F., Gruber, B. & Fründ, J. (2008). Introducing the bipartite package: analysing ecological networks. *R news*, 8/2, 8-11.

13.

Dormann, C.F. & Strauss, R. (2013). Detecting modules in quantitative bipartite networks: the QuaBiMo algorithm. *arXiv [q-bio.QM]*, 1304.3218.

14.

Fauna Europaea (2012). Fauna Europaea version 2.5. . Available at: <http://www.faunaeur.org>, last accessed 09 Sept 2013

15.

Gathmann, A., Greiler, H.J. & Tscharntke, T. (1994). Trap-nesting bees and wasps colonizing set-aside fields - succession and body-size, management by cutting and sowing. *Oecologia*, 98, 8-14.

16.

Henneman, M.L. & Memmott, J. (2001). Infiltration of a Hawaiian community by introduced biological control agents. *Science*, 293, 1314-1316.

17.

Hirao, T. & Murakami, M. (2008). Quantitative food webs of lepidopteran leafminers and their parasitoids in a Japanese deciduous forest. *Ecological Research*, 23, 159-168.

18.

Kaartinen, R. & Roslin, T. (2011). Shrinking by numbers: landscape context affects the species composition but not the quantitative structure of local food webs. *Journal of Animal Ecology*, 80, 622-631.

19.

Klein, A.M., Steffan-Dewenter, I. & Tscharntke, T. (2006). Rain forest promotes trophic interactions and diversity of trap-nesting hymenoptera in adjacent agroforestry. *Journal Of Animal Ecology*, 75, 315-323.

20.

Lewis, O.T., Memmott, J., Lasalle, J., Lyal, C.H.C., Whitefoord, C. & Godfray, H.C.J. (2002). Structure of a diverse tropical forest insect-parasitoid community. *Journal of Animal Ecology*, 71, 855-873.

21.

Macfadyen, S., Gibson, R., Polaszek, A., Morris, R.J., Craze, P.G., Planque, R.*, et al.* (2009). Do differences in food web structure between organic and conventional farms affect the ecosystem service of pest control? *Ecology Letters*, 12, 229-238.

22.

Memmott, J., Godfray, H.C.J. & Gauld, I.D. (1994). The structure of a tropical host-parasitoid community. *Journal of Animal Ecology*, 63, 521-540.

23.

Morris, R.J. unpublished data.

24.

Müller, C.B., Adriaanse, I.C.T., Belshaw, R. & Godfray, H.C.J. (1999). The structure of an aphid-parasitoid community. *Journal Of Animal Ecology*, 68, 346-370.

25.

Murakami, M., Hirao, T. & Kasei, A. (2008). Effects of habitat configuration on host-parasitoid food web structure. *Ecological Research*, 23, 1039-1049.

26.

Novotny, V., Miller, S.E., Baje, L., Balagawi, S., Basset, Y., Cizek, L.*, et al.* (2010). Guild-specific patterns of species richness and host specialization in plant-herbivore food webs from a tropical forest. *Journal of Animal Ecology*, 79, 1193-1203.

27.

Omacini, M., Chaneton, E.J., Ghersa, C.M. & Muller, C.B. (2001). Symbiotic fungal endophytes control insect host-parasite interaction webs. *Nature*, 409, 78-81.

28.

Paniagua, M.R., Medianero, E. & Lewis, O.T. (2009). Structure and vertical stratification of plant galler-parasitoid food webs in two tropical forests. *Ecological Entomology*, 34, 310-320.

29.

Ratnasingham, S. & Hebert, P.D.N. (2007). BOLD: The barcode of life data system ([www.barcodinglife.org)](http://www.barcodinglife.org)). *Molecular Ecology Notes* 7, 355-364.

30.

Roskov, Y., Kunze, T., Paglinawan, L., Orrell, T., Nicolson, D., Culham, A.*, et al.* (2013). Species 2000 & ITIS Catalogue of Life, 2013 Annual Checklist. Available at: [www.catalogueoflife.org/annual-checklist/2013/](http://www.catalogueoflife.org/annual-checklist/2013/), last accessed 09 Sept 2013

31.

Roslin, T. & Várkonyi, G. unpublished data.

32.

Rott, A.S. & Godfray, H.C.J. (2000). The structure of a leaf miner-parasitoid community. *Journal of Animal Ecology*, 69, 274-289.

33.

Sinclair, F.H. (2012). Community level consequences of adaptive management through Climate Matching: oak galls as a model system. PhD thesis. University of Edinburgh, U.K.

34.

Tscharntke, T., Gathmann, A. & Steffan-Dewenter, I. (1998). Bioindication using trap-nesting bees and wasps and their natural enemies: community structure and interactions. *Journal Of Applied Ecology*, 35, 708-719.

35.

Tylianakis, J.M., Tscharntke, T. & Lewis, O.T. (2007). Habitat modification alters the structure of tropical host-parasitoid food webs. *Nature*, 445, 202-205.

36.

Várkonyi, G. & Roslin, T. (2013). Freezing cold yet diverse – dissecting a high-Arctic parasitoid community associated with lepidopteran hosts. *The Canadian Entomologist*, special issue 2, 193-218.
